# Health Risk Assessment of Nail Technicians in the Formal and Informal Sectors of Johannesburg, South Africa

**DOI:** 10.3390/ijerph22030330

**Published:** 2025-02-24

**Authors:** Goitsemang Keretetse, Gill Nelson, Derk Brouwer

**Affiliations:** 1 School of Public Health, Faculty of Health Sciences, University of the Witwatersrand, Johannesburg 2193, South Africa; gill.nelson@wits.ac.za (G.N.); derk.brouwer@wits.ac.za (D.B.); 2 Department of Neurology, Barrow Neurological Institute, Phoenix, AZ 85013, USA

**Keywords:** volatile organic compounds, formaldehyde, self-reported symptoms, probabilistic risk assessment, Monte Carlo simulation

## Abstract

Exposure to volatile organic compounds (VOCs) in nail salons poses risks of acute and chronic health effects for nail technicians. The objectives of this study were to investigate differences in VOC exposure and self-reported symptoms among formal and informal nail technicians and assess the non-carcinogenic and carcinogenic risks using a probabilistic approach. A questionnaire was administered to 54 formal and 60 informal nail technicians to elicit information on sociodemographic characteristics, work conditions, and self-reported symptoms. Passive sampling was employed to measure 60 personal breathing zone concentrations of VOCs among 20 nail technicians (both formal and informal) over three consecutive days, along with 29 passive samples for formaldehyde in the informal sector. All VOC concentrations, except formaldehyde, were below occupational exposure limits. Sixty percent of the informal nail technicians and fifty-two percent of the formal nail technicians reported health-related symptoms; however, the difference was not statistically significant (*p* > 0.05). The median and 95th percentile non-cancer risks exceeded the hazard coefficient for 2-propanol in all technicians and MMA among informal technicians. The benzene and formaldehyde cancer risk estimates (medians and 95th percentiles) exceeded the US Environmental Protection Agency cancer risk threshold of 1 × 10^−6^. These findings indicate that nail technicians are at risk of developing acute and chronic health effects from long-term low-level exposure to VOCs.

## 1. Introduction

Nail technicians are exposed to volatile organic compounds (VOCs) emitted from nail products. Exposure is associated with the type and duration of nail applications, products used, working conditions, and number of clients serviced, among other factors. Nail services and applications include basic manicures and pedicures, nail polish, ultraviolet gel, acrylic nails, and artificial nails. Various products containing a cocktail of chemical ingredients are used for different purposes throughout each nail application. The chemicals include solvents such as aromatics hydrocarbons (toluene, ethyl benzene, xylene), aldehydes (formaldehyde), esters (ethyl acetate, butyl acetate, vinyl acetate, methyl methacrylate (MMA), and ethyl methacrylate (EMA)), ketones (acetone and methyl ethyl ketone), alcohols (ethanol, isopropyl alcohol, and n-butyl alcohol), plasticizers (dibutyl phthalate, triphenyl phosphate), and turpentines (pinene, limonene, camphor, menthol) [1,2,3,4]. All treatments involve the use of acetone. Nail polish contains several chemicals, such as lacquer, plasticizers, glosses, and hardeners. Some nail polishes may contain harmful substances commonly referred to as the ‘toxic trio’ [5,6], viz. toluene, dibutyl phthalate (DBP), and formaldehyde. Acrylic nails are made by combining polymer (a powder) and monomer liquid to form a paste. The monomer is made of MMA, a chemical substance that was banned in the US and other countries by the Food and Drug Administration due to its sensitizing properties in 1974 [7]. Although MMA was subsequently replaced by EMA, studies have since reported the presence of MMA in nail salons [4,8,9,10,11,12,13] in several countries. Some nail polish brands have added labels reporting the exclusion of certain toxic ingredients, such as lead [6,14] and the ‘toxic trio’; however, some of these substances, e.g., toluene, are still found in products in nail salons [11,15].

Salon characteristics, e.g., location, room volume, and ventilation, play a role in VOC concentrations. Studies have reported higher emissions of VOCs from nail salons without ventilation compared to those with ventilation [15,16]. Goldin et al. [17] measured total VOC (TVOC) concentrations, particulate matter of diameter less than 2.5 µm (PM2.5), and carbon dioxide in nail salons in Boston, USA, and found that nail services (e.g., pedicure, manicure, silk nails, gel nails, acrylic nails, waxing, and airbrushing) increased air pollutant concentrations and that ventilation improved indoor air quality. In Iran, salon activities were reported to be the source of VOCs due to the high inside-to-outside ratio of VOC concentrations in a study conducted by Hadei et al. [18]. They found significant relationships between the concentrations of some compounds (benzene, toluene, ethylbenzene, and formaldehyde) and the number of nail treatments; adequate ventilation reduced concentrations [18].

Nail technicians have reported symptoms such as headaches, lightheadedness, irritation of the eyes, skin, and respiratory system, and allergic sensitization [8,9,11,19,20,21]. In other studies, nail technicians reported more health symptoms than office workers [1,8,13,22]. In a study by Ma et al. [23], nail technicians reported experiencing symptoms since they started working in the nail industry, stating that the symptoms worsened at work. In another qualitative study, nail technicians identified health issues that they perceived to be directly related to workplace exposures, which included symptoms due to contact with nail products and poor ergonomics [24].

Although acute health effects related to chemical exposures in the nail care industry have been studied, there are only a few studies on the long-term effects of VOCs from nail products [11,18,25]. Benzene, toluene, ethylbenzene, and xylene are known to cause adverse health effects, such as cancer and neurological effects. Benzene has been confirmed to be carcinogenic to humans (Group 1) by the International Agency for Research on Cancer (IARC) and a known human carcinogen (Group A) by the US Environmental Protection Agency (EPA) [26,27]. Formaldehyde is classified as a probable carcinogen (Group B1) by the EPA’s Integrated Risk Information System (IRIS) and as a human carcinogen (Group 1) by the IARC [28,29].

We conducted a study to investigate differences in VOC exposure and self-reported symptoms in formal and informal nail technicians. We also assessed the non-carcinogenic and carcinogenic effects of selected VOCs using a probabilistic risk assessment approach.

## 2. Materials and Methods

### 2.1. Study Area and Study Design

This cross-sectional study was conducted in the City of Johannesburg, South Africa. The study participants comprised 60 nail technicians working in 60 conveniently selected informal nail salons in the Braamfontein area near the Johannesburg Central Business District and 54 nail technicians working in 14 conveniently selected formal nail salons located in the northern suburbs of Johannesburg. The formal nail salons were licensed and registered as part of a large franchise. Informal nail salons are not licensed or formally registered with any enterprise or establishment [30]. The nail salons offered basic nail services, including manicures and pedicures, nail polish, and gel and acrylic applications. The informal nail salons were nested in hair salons opening to the roadside. In contrast, the formal nail salons were located either in a shopping center/strip mall with the entrance opening to a carpark or inside a shopping mall with a central walkway where retail stores face one another. The mean room volume of the formal nail salons was almost twice that of the informal nail salons. Informal nail salons relied predominantly on natural ventilation through open doors (there were no windows). Some of the formal nail salons were equipped with mechanical ventilation in the form of heating, ventilation, and cooling (HVAC) systems. None of the participating salons had local extraction ventilation (LEV) systems. Further details about the nail technicians and the nail salons are described in a previously published paper [31].

The study was explained to all the nail technicians in the nail salons that were visited. Ethical approval was obtained from the University of the Witwatersrand Human Research Ethics Committee (HREC): certificate number M171184—approval date 20 November 2018.

### 2.2. Questionnaires

A questionnaire (Supplementary Table S1) was designed to collect data on sociodemographic characteristics, work experience, work practices, services offered, working hours, and health symptoms within the last four weeks. The questions were adapted from questionnaires used in other studies on nail technicians [1,8,20] and were piloted on 10 formal and 10 informal nail technicians [30] who did not take part in the main study. The questionnaire was in English, as the participants understood and were able to communicate in English. Trained research assistants administered the questionnaire before the start of the workday and/or between serving clients at the salon where the nail technicians worked.

### 2.3. Personal Exposure Assessment

Personal breathing zone concentrations were measured on a subset of the 114 participants, following the methodology described by Keretetse et al. [31]. A convenience sample of 20 participants (10 formal and 10 informal nail technicians) was selected to participate in the exposure assessment phase. A total of 60 personal breathing zone samples for VOCs were collected from 20 nail technicians (both formal and informal) over three consecutive days. Additionally, 29 passive samples for formaldehyde were collected from the informal nail technicians’ breathing zones.

Personal breathing zone samples were collected by diffusion/passive sampling (Radiello^®^ passive sampler, Sigma Aldrich, Kempton Park, South Africa) for VOC measurements, while formaldehyde was measured using an SKC UMEx 100 passive sampler (UMEx 100, SKC, Kempton Park, South Africa).

### 2.4. Chemical Analysis

Details of the VOC analysis have been published elsewhere [31]. For the analysis of formaldehyde, the OSHA 1007 analytical method was followed. Formaldehyde was extracted from the UMEX 100 samplers with 2 mL of high-purity acetonitrile using a Waters extraction manifold operated at 457 mmHg. Extracted samples were then diluted to 5 mL with additional acetonitrile and analyzed using an Infinity High-Pressure Liquid Chromatograph (HPLC) system equipped with a Phenomenex Luna 5 µm C18 100A (250 × 4.6 mm). The diode array detector was operated at 360 nm. Formaldehyde concentrations were calculated using standard calibration curves and the total volume of air sampled [18]. A field blank, stored alongside the formaldehyde samples, was used for blank correction. Chemical analysis was conducted by a SANAS-accredited laboratory using calibrated instruments and standardized analytical methods.

Based on the literature review, information obtained from the product labels, and Safety Data Sheets (SDSs), 20 VOCs were analyzed. To simplify the calculation of the average concentration, the limit of detection (LoD) for all VOCs was set at 0.50 µg/mL, and the average lowest concentration value of the samples was set at 0.01 mg/m^3^. The LoD for formaldehyde was 0.1 µg per sample.

### 2.5. Risk Assessment

A risk assessment was conducted to estimate the potential risk of exposure to VOCs from the nail treatments the nail technicians performed. The cancer risk from exposure to benzene and formaldehyde and the non-cancer risk from exposure to five selected VOCs (benzene, toluene, xylene, 2-propanol, and MMA) were estimated. Several studies have used a deterministic approach, which considers a point estimate of the worst-case scenario [11,32,33]. We adopted a probabilistic risk assessment approach, which estimates the probabilistic distribution of a risk function by treating some variables as random variables drawn from their respective distributions [18,34,35,36,37,38].

To calculate the inhalation risk assessments, we first calculated the adjusted air concentration (C_air-adj_) of each VOC to represent continuous exposure. The C_air-adj_ may be estimated as shown in Equation (1). For non-carcinogenic risks, the air concentration is adjusted based on the time over which exposure occurs (i.e., the exposure duration). For carcinogenic risks, the concentration is averaged over the lifetime of the exposed individual.

Equation (1):

(1) $$C_{air\text{-}adj}=\frac{C_{air}\times ET\times EF}{24\;hrs\times 365\;days}$$

where C_air_ is the concentration of contaminant in air (µg/m^3^), ET is the exposure time (hours/day), and EF is the exposure frequency (days/year) [39]. Based on the sampling data and responses to the questionnaires administered to the nail technicians, ET was the shift duration. The EF was determined to be 245 and 294 days per year for formal and informal nail technicians, respectively. This was based on an average working week of five days/week for formal nail technicians and six days/week for informal nail technicians, with both groups taking three weeks of vacation in a year.

The hazard quotient (HQ) was used to estimate the non-carcinogenic risk of VOCs (Equation (2)). An HQ greater than one is regarded as the potential for adverse health effects, whereas an HQ value of less than one indicates that adverse effects are unlikely to occur.

Equation (2):

(2) $$HQ=\frac{C_{air\text{-}adj}}{RfC}$$

where HQ is the hazard quotient of VOC species, C_air-adj_ is the adjusted air concentration (µg/m^3^) calculated using Equation (1), and RfC is the reference concentration (µg/m^3^) obtained from the USEPA IRIS [40].

The cancer risk for carcinogenic compounds was calculated using Equation (3):

(3) $$CR=IUR\times C_{air\text{-}adj}\times \frac{ED}{AT}$$

where IUR is the inhalation unit risk (µg/m^3^) for the pollutant of interest, as shown in Table 1; C_air-adj_ is the adjusted air concentration (µg/m^3^) as calculated in Equation (1); ED is the exposure duration (years); and AT is the average lifetime (years). The exposure duration was assumed to be 30 years, which is a typical work lifetime among nail technicians [18,25].

### 2.6. Probabilistic Assessment and Sensitivity Analysis

To address the variability for each parameter in the risk assessment, a probabilistic approach was used. We applied ModelRisk (Vose Software version 6.3.2) to simulate the distribution of the risk calculation outputs, i.e., HQ or CR values. The variables used and their distributions are listed in Table 1. It should be noted that AT used in Equation (3) was specific to the South African context and differed by sex, whereas other parameters were retrieved from the EPA Exposure Factors Handbook and our study. All variables were randomly entered into a Monte Carlo simulation with 10,000 iterations. A sensitivity analysis was conducted using Crystal ball software (OCB10BSL-EN) (Oracle, classroom) to identify the parameters that significantly affected the health risk assessment outcome. The simulation outputs are presented using the 50th percentile and 95th percentile values, and a histogram and a sensitivity (tornado) plot are presented for each outcome.

### 2.7. Data Analysis

Data pre-processing, including data cleaning and visualization, was conducted using Microsoft Excel 2016. Descriptive statistics, including (geometric) means, (geometric) standard deviations, and interquartile ranges, were calculated using ExpoStats (version 1.0), a Bayesian Toolkit [44], and Stata software (version 17). Imputation for data below the limit of quantification (LoQ) was performed using the Expostats-NDexpo/RoS tool (version 1.0). NDexpo implements a rigorous censored data treatment method by labeled regression on order statistics [45]. For the data in this study, the analytical LoQ was converted into a LoQ sampling concentration of 0.01 mg/m^3^.

Self-reported health symptoms were reported as binary answers (yes/no), and results are reported as percentages of participants responding ‘yes’ to each question. The Welch *t*-test was used to compare self-reported symptoms between formal and informal nail technicians. The self-reported symptoms were categorized into four categories: neurological effects (category A), respiratory effects (category B), eye irritation (category B), and skin irritation (category D) (Supplementary Table S2). The symptoms score was calculated using Equation (4). This was applied to all symptom categories except for skin symptoms, which are not related to inhalation exposure.

Equation (4):

(4) $$\sum_{Ni}^{ni}symptomscore=\frac{nA}{NA}+\frac{nB\;}{NB}+\frac{nC\;}{NC}+\frac{nD}{ND}$$

where *ni* is the number of self-reported symptoms in that category, and *Ni* is the total number of symptom options.

Self-reported symptom data (symptom categories and symptom score) from the questionnaires were correlated with exposure factors, including VOC concentrations and demographic data. The statistical package JASP (version 0.18.3) [46] was used to test the association between continuous variables. Spearman’s rho correlation was used for continuous variables, while the point biserial correlation analysis (r_pb_) was used for categorical variables such as sex. Concentrations of VOCs assigned to categories with similar health effects were summed (Supplementary Table S3).

To determine the respiratory additive adverse effect, the American Conference of Governmental Industrial Hygienists (ACGIH) additive mixture formula was used (Equation (5)) [47,48], similar to Alaves et al. [4]. Based on the reported respiratory effects, the chemicals of interest for formal nail technicians were ethanol, acetone, ethyl acetate, toluene, xylene, propyl acetate, n-butyl acetate, and 2-propanol. For informal nail technicians, these were ethanol, acetone, ethyl acetate, toluene, xylene, MMA, benzene, and formaldehyde.

Equation (5):

(5) $$\sum_{i=1}^{n}\frac{C_{i}}{T_{i}}=\frac{C_{1}}{T_{1}}+\frac{C_{2}}{T_{2}}+\frac{C_{3}}{T_{3}}+\ldots +\frac{C_{n}}{T_{n}}\leq 1\;$$

where *Ci* is the measured concentration of a particular chemical in the mixture, and *Ti* is the corresponding threshold limit for that chemical. In the additive mixing formula, ‘1’ represents the exposure threshold limit for the mixture. If the equation generates a result > 1, then the exposure limit for the mixture has been exceeded. The occupational exposure limit (OEL) value used was the South African 8 h time-weighted average (TWA) OEL.

## 3. Results

### 3.1. Demographic Characteristics of Study Participants

Table 2 presents the demographic characteristics of the nail technicians. All formal nail technicians were female, while 26 (43.3%) of the informal nail technicians were male. On average, the formal sector nail technicians were older (33.11 ± 6.41) and had worked longer in the nail industry (5.07 ± 3.71) than the informal nail technicians. Although the informal nail technicians worked longer hours per day on average (9.22 ± 1.03) than the formal nail technicians (8.67 ± 0.95), they serviced fewer clients per day (4.80 ± 1.44) than the formal nail technicians (6.72 ± 1.82).

**Table 2:** Demographic characteristics of formal and informal nail technicians (N = 114).

	Formal Nail Technicians (*n* = 54)					Informal Nail Technicians (*n* = 60)				
Characteristic	*n*	%	AM	SD	Range	*n*	%	AM	SD	Range
Age (years)			33.11	6.41	23–57			30.12	4.75	22–44
Sex										
Male	0	-				26	43.3			
Female	54	100				34	56.7			
Work experience (years)			5.07	3.71	1–18			3.28	1.96	1–7
No. clients serviced per day			6.72	1.82	3–10			4.80	1.44	2–9
No. working hours per day			8.67	0.95	8–12			9.22	1.03	8–12
No. working days per week			5.17	0.80	4–7			6.42	0.53	5–7

*n*, sample number; %, percentage; AM, arithmetic mean; SD, standard deviation.

### 3.2. Characterization of Volatile Organic Compounds

The GM concentrations of acetone, ethyl acetate, and ethanol were significantly higher among formal nail technicians than informal nail technicians. Noticeably, a 100% DF was observed for n-butyl acetate among the formal nail technicians, whereas it was detected in only one of the twenty-nine samples (3.4%) from the informal nail technicians. Methyl methacrylate was found in 89.7% of the samples among the informal nail technicians but in one sample (3.4%) among the formal nail technicians. Formaldehyde concentrations were below the LoD for the formal nail salons; therefore, only formaldehyde concentrations from the informal nail salons were analyzed. Table 3 summarizes the VOC concentrations that were included in the analysis. Except for formaldehyde, all the VOC concentrations were well below their respective SA 8 h TWA OEL, as well as the OSHA compliance standards for permissible exposure limits (PELs) and the ACGIH recommended threshold limit values (TLVs). However, the calculated additive mixture threshold (from Equation (5)) was exceeded in all the informal nail salons.

**Table 3:** Summary of VOC concentrations (mg/m^3^) in the formal and informal nail technicians (table adapted from Keretetse et al. [31]).

		Formal Nail Salons (*n* = 29)						Informal Nail Salons (*n* = 29)						
VOC	*n* (%)	AM	SD	GM	GSD	IQR	*n* (%)	AM	SD	GM	GSD	IQR	SA OEL	ACGIH TLV
Ethanol	29 (100)	4.24	1.75	3.87	1.58	3.16–5.01	28 (96.6)	6.61	12.30	2.35	4.4	1.19–5.50	1900	1880
Acetone	29 (100)	170.2	238.03	108	2.49	64.69–162.22	29 (100)	50.53	46.26	24.3	5.11	11.3–78.7	1200	594
Ethyl acetate	29 (100)	2.35	1.50	2.01	1.72	1.46–3.13	24 (82.8)	0.81	1.88	0.21	5.4	0.08–0.58	2900	1440
Benzene	5 (17.2)	0.02	0.003	0.02	1.12	0.019–0.023	11 (37.9)	0.02	0.01	0.02	1.46	0.01–0.02	3	1.6
MMA	1 (3.4)	0.02	0.02	0.02	1.48	0.017–0.017	26 (89.7)	41.66	73.78	8.06	9.48	2.41–54.45	410	205
EMA	26 (86.7)	7.21	13.84	1.56	6.93	0.39–5.67	24 (82.8)	30.89	51.37	3.57	14.8	0.35–32.81	^b^	^b^
Propyl acetate	18 (62.1)	0.06	0.07	0.04	2.23	0.026–0.068	3 (10.3)	0.01	0.02	0.02	2.23	0.01–0.08	840	417
Toluene	28 (96.6)	0.05	0.04	0.04	1.73	0.03–0.06	26 (89.7)	0.04	0.02	0.03	1.47	0.02–0.04	151	75
n-Butyl acetate	29 (100)	0.78	0.55	0.62	1.97	0.41–0.96	1 (3.4)	0.02	0.019	0.02	1.45	0.02–0.12	475	238
Xylene	15 (51.7)	0.04	0.03	0.03	1.88	0.022–0.043	23 (79.3)	0.03	0.02	0.03	1.49	0.02–0.03	868	87
d-Limonene	28 (96.6)	0.24	0.30	0.14	2.68	0.09–0.21	13 (44.8)	0.10	0.2	0.02	6.99	0.01–0.08	^b^	^b^
2-Propanol	28 (96.6)	49.9	25.9	43.7	1.72	33.79–57.95	7 (24.1)	1.38	2.43	0.34	6.16	0.099–1.02	983	492
White spirits	24 (82.7)	1.09	1.45	0.44	4.81	0.10–1.19	27 (93.1)	1.26	0.99	0.9	2.45	0.49–1.67	^b^	^b^
Formaldehyde	n.a	n.a	n.a	n.a	n.a	n.a	29 (100)	0.21	0.05	0.21	1.24	0.181–0.213	0.2	0.12
^a^ Ʃ Ci/Ti		0.196	0.133	0.17	1.73	0.103–0.216		1.21	0.29	1.18	1.25	1.03–1.33	1	1

*n*, sample number; %, detection frequency; AM, arithmetic mean; SD, standard deviation; GM, geometric mean; GSD, geometric standard deviation; IQR, interquartile range; SA OEL, South African occupational exposure limit; MMA, methyl methacrylate; EMA, ethyl methacrylate; n.a, not analyzed. ^a^ Ʃ Ci/Ti, ACGIH additive effect (respiratory effect). ^b^ Level was not established for ethyl methacrylate, d-limonene, and white spirit.

### 3.3. Self-Reported Symptoms and Exposure Factors

Table 4 summarizes the acute health-related symptoms reported over the past four weeks. Sixty percent (*n* = 36) of the informal nail technicians reported having health-related symptoms compared to 52% (*n* = 28) of the formal nail technicians. The difference, however, was not statistically significant (*p* = 0.381). Of the latter group, 17% experienced one symptom, while 35% experienced multiple symptoms. For the informal nail technicians, 12% experienced one symptom, and 48% experienced multiple symptoms across the different symptom categories. Informal male nail technicians reported more symptoms (69%) than their female counterparts (53%). Some symptoms were perceived to be caused by specific nail products or were related to specific nail applications. The difference in the mean number of reported symptoms between the two groups of nail technicians was not statistically significant.

The Spearmen’s (rho) and the point-biserial correlation (r_pb_) coefficients are shown for symptom categories, corresponding VOC categories, and selected exposure variables in Supplementary Table S4. A significant correlation was found between age and respiratory effects (rho = −0.814, *p* = 0.004) and sex and neurological effects for informal nail technicians (rho = −0.745, *p* = 0.013). There were statistically significant correlations between working hours per day and respiratory effects (r_pb_ = −0.931, *p* = <0.001) and between the symptom score and working hours (r_pb_ = −0.657, *p* = 0.039) among the informal nail technicians.

### 3.4. Cancer and Non-Cancer Risk Estimation

The VOCs investigated in this study are not reported to have carcinogenic effects, except for benzene and formaldehyde [26,27,28,29]. Only benzene, toluene, xylene, 2-propanol, and MMA were considered for the non-cancer risk characterization. Acetone and formaldehyde were not considered because no reference concentration has been established to date, as per the US EPA guidelines [29,49]. The median and 95th percentile non-cancer risks were above one for 2-propanol for formal and informal nail technicians; the same was found for MMA among the informal nail technicians. These results are indicative of a probability of developing adverse health effects (Table 5).

The estimated increase in lifetime cancer risk for nail technicians in all nail salons based on 30 years of exposure is also presented in Table 5. Formaldehyde was not assessed for the formal female nail technicians because the concentration levels were below the LoD. The estimated cancer risk values (medians and 95th percentiles) for benzene and formaldehyde exceeded the US EPA risk level of 1 × 10^−6^. The median lifetime cancer risk for formal female nail technicians for benzene was 1.73 × 10^−5^. For informal male nail technicians, the median lifetime cancer risks for benzene and formaldehyde were 2.28 × 10^−5^ and 3.38 × 10^−4^, respectively.

**Figure 1:**
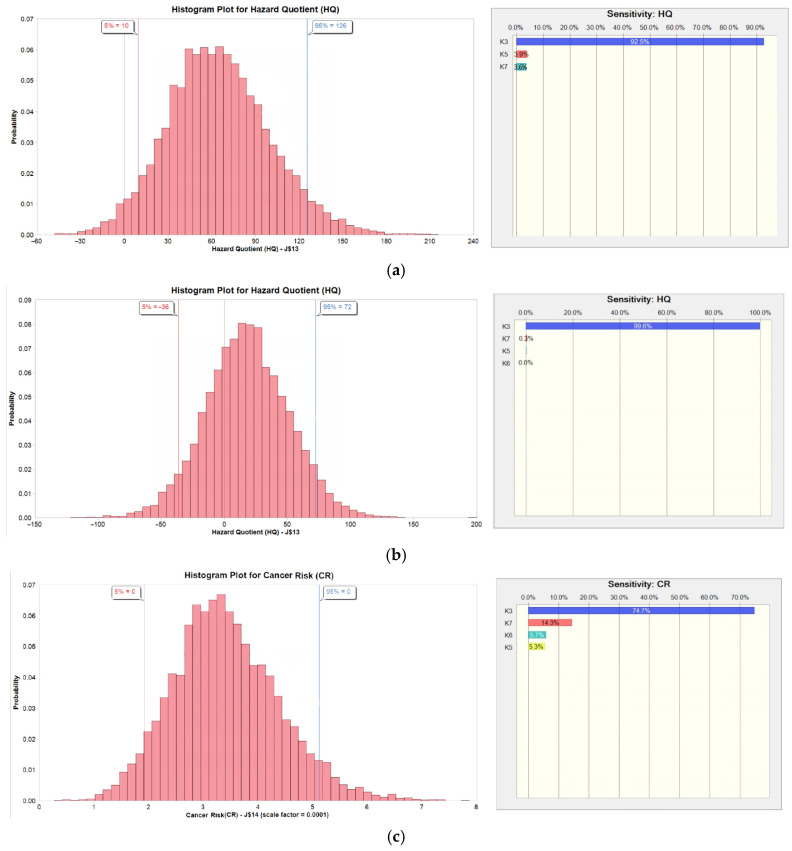
Probabilistic distribution and sensitivity analysis of estimated non-cancer risk assessment hazard quotient (HQ) for (**a**) 2-propanol among formal female nail technicians and (**b**) MMA among informal female nail technicians and (**c**) estimated cancer risk assessment (CR) for formaldehyde among informal male nail technicians.

The results of the sensitivity analysis of the non-cancer and cancer risk assessment are represented in Table 6 and Figure 1. The C_air-adj_ variations dominated cancer and non-cancer risk outcome variables. This is expected considering the wide variation of the C_air-adj_ versus the narrow distribution of the exposure frequency (EF) and exposure time (ET).

## 4. Discussion

In this study, we investigated differences in VOC exposure and self-reported symptoms in formal and informal nail technicians. We also estimated the non-carcinogenic and carcinogenic risks associated with these exposures.

Although informal nail technicians’ working hours were longer than those of formal nail technicians, the latter group serviced more clients and, therefore, performed more nail applications. To speed up the process, as described by Keretetse et al. [31], the formal nail technicians used a quick and easy method to remove existing nail applications by immersing clients’ hands in an acetone hot bath. The informal nail technicians commonly used cotton wool soaked in acetone—a slower method requiring less acetone. As reported in the earlier study by Keretetse et al. [31], acetone concentrations were significantly higher in the formal than the informal nail salons.

More of the formal than the informal nail technicians reported ill health symptoms (60% and 52%, respectively); however, the difference was not statistically significant (*p* > 0.05). The most frequently reported symptoms were headaches, eye irritation, chest tightness, nasal congestion, and skin problems. Other studies have reported similar findings, viz. headaches, confusion, skin irritation, eye irritation, and nose irritation, as well as other symptoms such as musculoskeletal disorders (e.g., neck, back, and wrist pain). Although our study participants reported similar musculoskeletal-related symptoms, these are not presented here.

All three symptom categories included in the current study, namely respiratory, neurological, and eye irritation, were negatively correlated with their respective VOC categories for all nail technicians. This could be due to a systematic bias, whereby the symptoms reported may not have been related to the exposure concentrations during the assessment days. Acute symptoms may be related to peak exposures rather than daily averaged full shift exposure concentrations [50,51]. Moreover, as shown in Table S2, there was an overlap of VOCs assigned to the different symptom categories, which reduced the resolution between the categories. For formal nail technicians, no other variables were significantly correlated with symptoms. For the informal nail technicians, however, a statistically significant correlation existed between respiratory symptoms and the number of hours worked per day. A similar correlation was found for symptom scores. A study conducted in New York and New Jersey by Seo et al. [52], following the implementation of local exhaust ventilation (LEV) regulations in New York, showed a higher proportion of New York nail salons having installed mechanical ventilation and LEV systems, as well as more frequent use of PPE, compared to nail salons in New Jersey. Despite the reported presence of the LEV system and the use of personal protective measures, a significant proportion of participants reported experiencing health-related concerns and symptoms similar to those reported in the current study.

Task-based measurements conducted by the same team [31] showed high peak exposures during nail applications, including soak-off to remove existing nail polish. Acrylic applications emitted higher levels of TVOC concentrations than other nail applications. To save time, some of the nail applications were conducted simultaneously on the client’s hands and feet, but these increased exposure levels.

The MMA concentrations in the informal nail salons are also related to acrylic nail applications, which are more popular than in the formal sector. All VOCs were compared to compliance standards and were below their respective exposure limits, except formaldehyde, which exceeded both the South African 8 h TWA and the ACGIH TLV. The ACGIH, mixed additive effects index for respiratory effects, was exceeded in informal nail salons, probably due to the formaldehyde concentration exceeding its exposure limit. These findings are comparable with those described by Alaves et al. [4]. Formaldehyde, a known carcinogen (IARC) and one of the toxic trio, can still be found as an ingredient in some nail polishes and nail hardeners globally. It may also be emitted from building materials and other products, such as those used for hairdressing activities. Many of the informal nail salons in this study included hairdressing activities. The higher formaldehyde levels may have emanated from hairdressing activities in informal nail salons.

The VOC concentrations (Table 3) were, in general, less variable in the formal than in the informal sector. This was partly due to the number of clients treated per work hour, i.e., 0.77 versus 0.52 in the formal and informal sectors, respectively, but also to the contribution of VOC emissions from co-workers’ activities, as mentioned. This ‘ bystander exposure’ dampens the fluctuations of concentrations and was not observed in the informal sector since only one technician was providing services at a time [31]. In the formal nail salons, nail services were performed by co-workers, and associated VOC emissions contributed to the relatively high TVOC concentrations.

Non-carcinogenic and carcinogenic risk assessments were estimated for selected VOCs using the hazard quotient and cancer risk for inhalation exposure. The mean concentration for VOCs was used in the calculation, following the consensus to use the arithmetic mean rather than the geometric mean for calculating contaminant concentration levels for risk assessment purposes. An individual’s long-term average exposure is better presented as an arithmetic mean. The use of the geometric means would have reduced the impact of any high values of the measured concentrations [53].

Since the RfC is for 24 h exposures, a direct comparison of the RfCs with the mean VOC concentrations over the working hours (Table 3) is inappropriate. Instead, an indirect comparison of the VOC concentrations with the RfC was adopted, i.e., by averaging the mean concentration for the working hours over a 24 h day. The hazard quotient compares the averaged 24 h intake of pollutants with the intake resulting from 24 h exposure at the RfC concentration level. The 95th percentile, which is considered a worst-case scenario, exceeded the non-cancer risk (HQ) of 1 (unacceptable risk) for 2-propanol for the formal nail technicians and for both MMA and 2-propanol for the informal nail technicians. For the formal female nail technicians, 2-propanol posed the highest risk of all the VOCs. Although the average concentrations for 2-propanol over the workday were lower than the OEL, the mean concentration over 24 h exceeded the RfC. For the informal nail technicians, the same held for MMA concentrations, with the highest median and 95th percentile HQ estimated values of 1.84 × 10^1^ and 7.23 × 10^1^, respectively. Similar findings were reported by Tran et al. [22], where the mean MMA non-cancer risk was above 1, and the concentration was eight times higher than the RfC. These findings may be related to the type of nail products used and the frequency of nail applications, with the acrylic application method used more frequently in the informal sector. All estimated cancer risk values (i.e., medians and 95th percentiles) for benzene and formaldehyde exceeded the US EPA risk level of 1 × 10^−6^, which concurred with findings reported in other nail salon studies [11,18,22,25]. The sensitivity analysis showed that the variation in the adjusted air concentration of the VOCs was the main contributor to the variations in cancer and non-cancer risk outputs.

There were several limitations in this study. The relatively small sample size and convenient sampling of the participants made it challenging to extrapolate the findings to all nail technicians in Johannesburg or South Africa. We only considered inhalation as the route of entry and not dermal exposure. We relied on self-reported symptoms to determine the acute health effects of exposure to VOCs in nail salons. The time difference between administering the questionnaires and taking measurements may have affected the association between the reported symptoms and VOC exposures since the participants were not asked to report symptoms at the end of each measurement day. Traffic-related pollutants could infiltrate nail salons, particularly those that are open to the roadside. However, real-time direct-reading measurements taken both inside and outside the salon indicated that these levels were very low. Product information was not collected at all participating nail salons, and we relied on the published literature for the information and VOC content of commonly used nail products.

Despite these limitations, we generated personal breathing zone concentration data for nail salon workers, including those in the informal sector, which are always difficult to include in studies like this. We also found differences between the two sectors related to salon characteristics, working conditions, types of nail applications provided, and self-reported symptoms. Although the VOC concentrations were below their respective OELs, the non-cancer risk estimates exceeded the HQ of 1, which indicated that continued exposure may lead to long-term adverse health effects. The lifetime cancer risk was higher than the recommended US EPA risk level (1 × 10^−6^) for benzene and formaldehyde. This represents a cancer risk resulting from exposure to these compounds. Similar findings were reported in a study conducted in nail salons in Colorado, USA [11].

## 5. Conclusions

This study contributes to the research on nail technicians’ exposure to various VOCs emitted from nail products used daily. There were differences in age, sex, and working conditions between the formal and informal nail technicians. The informal nail technicians reported more ill health symptoms than the formal nail technicians. The most common symptoms were headaches, eye irritation, respiratory effects, and skin disorders. Informal male nail technicians reported more symptoms than their female counterparts.

Although exposure levels for the informal nail technicians were below the recommended OELs for the selected VOCs, except for formaldehyde, the findings suggest a risk of developing both carcinogenic and non-carcinogenic health effects. Among both the formal and informal nail technicians, the estimated non-cancer risks for MMA and 2-propanol were higher than the acceptable limit of 1, indicating a risk of developing adverse health effects. All the estimated cancer risk levels for benzene and formaldehyde exceeded the EPA cancer risk threshold of 1 × 10^−6^. The sensitivity analysis for MMA, 2-propanol, and formaldehyde showed that adjusted air concentration contributed the most to the variation of the cancer and non-cancer risk outputs. Efforts to reduce exposure and regulate working hours, thereby reducing the frequency of exposure, should be made to protect nail technicians from developing adverse health effects from long-term exposure to VOCs. Control measures should be applied following the hierarchy of controls. Substituting hazardous chemicals with safer alternatives is essential. Engineering controls, such as local exhaust ventilation and downdraft tables, can significantly lower airborne emissions. Administrative controls, including safer work practices, job rotation, and PPE, can be implemented to further protect workers. The ‘soak-off’ nail polish removal method should also be re-evaluated, weighing its time-saving benefits against the risk of high acetone exposure. Research should be conducted to estimate the incidence of chronic health effects associated with exposure to VOCs and other vapors and dusts emitted during the course of their work in longitudinal cohort studies. Chemicals in nail products should be linked to specific nail applications and the measured exposures in nail salons. This will inform policies regarding the identification of hazardous chemical ingredients and the regulation of specific chemicals, including the potential banning of the use of some chemicals in nail products.

## Figures and Tables

**Table 1:** Variables used for cancer and non-cancer risk assessments among formal and informal nail technicians.

Parameter	Definition	Unit	Distribution	Value	Source
C_air_	Concentration of VOC in air	µg/m^3^	Normal	VOC concentrations *	Measured data
ET (formal NTs)	Exposure time	hours/day	Normal	8.67 ± 0.95 (formal NTs)	Questionnaire data
ET (informal NTs)	Exposure time	hours/day	Normal	9.22 ± 1.03 (informal NTs)	Questionnaire data
EF (formal NTs)	Exposure frequency	days/year	Triangle	245 (196–343)	Questionnaire data
EF (informal NTs)	Exposure frequency	days/year	Triangle	294 (245–343)	Questionnaire data
ED	Exposure duration	years	Triangle	30 (20–45)	U.S. EPA [41]
AT (female)	Average lifetime (life expectancy)	years	Fixed	68.4 (female)	Statistics South Africa [42]
AT (male)	Average lifetime (life expectancy)	years	Fixed	62.4 (male)	Statistics South Africa [42]
RfC	Inhalation reference concentration	µg/m^3^		5000 for toluene 100 for xylene 700 for MMA 200 for 2-propanol 30 for benzene	U.S. EPA [40] U.S. EPA [40] U.S. EPA [40] Burleigh-Flayer et al. [43] U.S. EPA [40]
IUR	Inhalation unit risk	µg/m^3^		7.8 × 10^−6^ for benzene 1.1 × 10^−5^ for formaldehyde	U.S. EPA [40] U.S. EPA [29]
C_air-adj_	Adjusted air concentration	µg/m^3^		Output	
CR	Cancer risk	unit-less		Output	
HQ	Hazard quotient (non-cancer risk)	unit-less		Output	

* See Table 3. MMA, methyl methacrylate; NTs, nail technicians, IUR, inhalation unit risk. Values are presented as mean ± SD and median (range).

**Table 4:** Self-reported symptoms among formal and informal nail technicians.

	Formal Nail Technicians (*n* = 54)	Informal Nail Technicians (*n* = 60)
Category	*n* (%)	*n* (%)
Category A (neurological effects)		
Headache	15 (27.8)	18 (30.0)
Light-headedness	8 (14.8)	6 (10.0)
Category B (respiratory effects)		
Difficulty breathing	7 (12.9)	5 (8.3)
Chest tightness	12 (22.2)	7 (11.7)
Regular cough	7 (12,9)	8 (13.3)
Wheezing	4 (7.41)	0 (0)
Nasal congestion	11 (20.4)	8 (13.3)
Doctor diagnosed asthma	1 (1.9)	0 (0)
Throat irritation	4 (7.4)	4 (6.7)
Nose irritation	7 (12.9)	13 (21.7)
Category C (eye irritation)	15 (27.8)	14 (23.3)
Category D (skin irritation)		
Skin problems	8 (14.8)	9 (15)
Eczema	2 (3.7)	3 (5.0)
Experienced symptoms	28 (51.9)	36 (60.0)
No symptoms experienced	26 (48.1)	24 (40.0)
Reported 1 symptom	9 (16.7)	7 (11.7)
Reported 2–5 symptoms	13 (24.1)	24 (40.0)
Reported ≥6 symptoms	6 (11.1)	5 (8.3)

**Table 5:** Health risk assessment for formal and informal nail technicians by Monte Carlo simulation.

		C_air-adj_ Percentile		HQ Percentile		CR Percentile	
Nail Technician Group	Pollutant	50th	95th	50th	95th	50th	95th
Formal female	Toluene	1.25 × 10^1^	3.07 × 10^1^	2.49 × 10^−3^	6.14 × 10^−3^	-	-
	Xylene	9.87	2.39 × 10^1^	9.87 × 10^−2^	2.39 × 10^−1^	-	-
	MMA	4.98	1.42 × 10^1^	7.12 × 10^−3^	2.02 × 10^−2^	-	-
	2-Propanol	1.26 × 10^4^	2.52 × 10^4^	**6.32 × 10^1^**	**1.26 × 10^2^**	-	-
	Benzene	5.07	7.21	1.69 × 10^−1^	2.40 × 10^−1^	**1.73 × 10^−5^**	**2.51 × 10^−5^**
	Formaldehyde	n.a	n.a	n.a	n.a	n.a	n.a
Informal female	Toluene	1.22 × 10^1^	2.34 × 10^1^	2.44 × 10^−3^	4.67 × 10^−3^	-	-
	Xylene	9.09	1.98 × 10^1^	9.09 × 10^−2^	1.98 × 10^−1^	-	-
	MMA	1.29 × 10^4^	5.06 × 10^4^	**1.84 × 10^1^**	**7.23 × 10^1^**	-	-
	2-Propanol	4.22 × 10^2^	1.69 × 10^3^	**2.11**	**8.43**	-	-
	Benzene	6.12	1.16 × 10^1^	2.04 × 10^−1^	3.88 × 10^−1^	**2.07 × 10^−5^**	**3.99 × 10^−5^**
	Formaldehyde	6.38 × 10^1^	9.53 × 10^1^	-	-	**3.08 × 10^−4^**	**4.66 × 10^−4^**
Informal male	Toluene	1.22 × 10^1^	2.33 × 10^1^	2.44 × 10^−3^	4.67 × 10^−3^	-	-
	Xylene	9.09	1.98 × 10^1^	9.09 × 10^−2^	1.98 × 10^−1^	-	-
	MMA	1.29 × 10^4^	5.06 × 10^4^	**1.84 × 10^1^**	**7.23 × 10^1^**	-	-
	2-Propanol	4.22 × 10^2^	1.69 × 10^3^	**2.11**	**8.43**	-	-
	Benzene	6.12	1.16 × 10^1^	2.04 × 10^−1^	3.88 × 10^−1^	**2.28 × 10^−5^**	**4.40 × 10^−5^**
	Formaldehyde	6.39 × 10^1^	9.53 × 10^1^	-	-	**3.38 × 10^−4^**	**5.12 × 10^−4^**

C_air-adj_, adjusted air concentration; HQ, hazard quotient (non-cancer risk); CR, cancer risk; MMA, methyl methacrylate; n.a, not analyzed. “-” indicates the HQ for non-cancer risk and CR for cancer risk were not calculated. Values in bold represent exceedance of the non-cancer risk and cancer risk threshold.

**Table 6:** Sensitivity analysis of estimated cancer risk assessment (CR) for formaldehyde among male nail technicians and the non-cancer risk assessment among female nail technicians.

		Contribution to Variance of the Output (%)			
Group	Output	C_air-adj_	EF	ET	ED
Formal female	HQ 2-propanol	92.5	3.9	3.6	n.a.
Informal female	HQ MMA	99.6	<0.1	0.3	n.a.
Informal male	CR formaldehyde	74.7	5.3	14.3	5.7

C_air-adj_, adjusted air concentration; EF, exposure frequency; ET, exposure time; ED, exposure duration; HQ, hazard quotient (non-cancer risk); CR, cancer risk; MMA, methyl methacrylate; n.a, not applicable.

## Data Availability

The data are available upon request to the corresponding author.

## Supplementary Materials

The following supporting information can be downloaded at https://www.mdpi.com/article/10.3390/ijerph22030330/s1, Table S1: Questionnaire; Table S2: Categories of self-reported symptoms; Table S3: Volatile organic compound categories according to the similarity of health effect; Table S4: Correlation coefficient and *p*-values between self-reported symptoms and VOC concentrations among formal and informal nail technicians.

## References

[1] Park S.A., Gwak S., Choi S. (2014). Assessment of occupational symptoms and chemical exposures for nail salon technicians in Daegu City, Korea. J. Prev. Med. Public Health.

[2] Ceballos D.M., Craig J., Fu X., Jia C., Chambers D., Chu M.T., Fernandez A.T., Fruh V., Petropoulos Z.E., Allen J.G. (2019). Biological and environmental exposure monitoring of volatile organic compounds among nail technicians in the Greater Boston area. Indoor Air.

[3] Craig J.A., Ceballos D.M., Fruh V., Petropoulos Z.E., Allen J.G., Calafat A.M., Ospina M., Stapleton H.M., Hammel S., Gray R. (2019). Exposure of Nail Salon Workers to Phthalates, Di(2-ethylhexyl) Terephthalate, and Organophosphate Esters: A Pilot Study. Environ. Sci. Technol.

[4] Alaves V.M., Sleeth D.K., Thiese M.S., Larson R.R. (2013). Characterization of indoor air contaminants in a randomly selected set of commercial nail salons in Salt Lake County, Utah, USA. Int. J. Environ. Health Res..

[5] OSHA (2017). Health Hazards in Nail Salons.

[6] Young A.S., Allen J.G., Kim U.-J., Seller S., Webster T.F., Kannan K., Ceballos D.M. (2018). Phthalate and Organophosphate Plasticizers in Nail Polish: Evaluation of Labels and Ingredients. Environ. Sci. Technol..

[7] U.S. FDA (2013). Nail Care Products. http://www.fda.gov/Cosmetics/ProductsIngredients/Products/ucm127068.htm.

[8] Harris-Roberts J., Bowen J., Sumner J., Stocks-Greaves M., Bradshaw L., Fishwick D., Barber C.M. (2011). Work-related symptoms in nail salon technicians. Occup. Med..

[9] Quach T., Gunier R., Tran A., Von Behren J., Doan-Billings P.A., Nguyen K.D., Okahara L., Lui B.Y.B., Nguyen M., Huynh J. (2011). Characterizing workplace exposures in Vietnamese women working in California nail salons. Am. J. Public Health.

[10] Grešner P., Stepnik M., Król M.B., Swiercz R., Smok-Pieniazek A., Twardowska E., Gromadzinska J., Wasowicz W. (2015). Dysregulation of markers of oxidative stress and DNA damage among nail technicians despite low exposure to volatile organic compounds. Scand. J. Work. Environ. Health.

[11] Lamplugh A., Harries M., Xiang F., Trinh J., Hecobian A., Montoya L.D. (2019). Occupational exposure to volatile organic compounds and health risks in Colorado nail salons. Environ. Pollut..

[12] Zhong L.X., Batterman S., Milando C.W. (2019). VOC sources and exposures in nail salons: A pilot study in Michigan, USA. Int. Arch. Occup. Environ. Health.

[13] Lteif M., El Hayek M.S., Azouri H., Antonios D. (2020). Assessment of work-related symptoms, perceived knowledge, and attitude among nail salon technicians. Toxicol. Ind. Health.

[14] Ceballos D.M., Young A.S., Allen J.G., Specht A.J., Nguyen V.T., Craig J.A., Miller M., Webster T.F. (2021). Exposures in nail salons to trace elements in nail polish from impurities or pigment ingredients—A pilot study. Int. J. Hyg. Environ. Health.

[15] Harrichandra A., Roelofs C., Pavilonis B. (2020). Occupational Exposure and Ventilation Assessment in New York City Nail Salons. Ann. Work. Expo. Health.

[16] Pavilonis B., Roelofs C., Blair C. (2018). Assessing indoor air quality in New York City nail salons. J. Occup. Environ. Hyg..

[17] Goldin L.J., Ansher L., Berlin A., Cheng J., Kanopkin D., Khazan A., Kisivuli M., Lortie M., Peterson E.B., Pohl L. (2014). Indoor air quality survey of nail salons in Boston. J. Immigr. Minor. Health.

[18] Hadei M., Hopke P.K., Shahsavani A., Moradi M., Yarahmadi M., Emam B., Rastkari N. (2018). Indoor concentrations of VOCs in beauty salons; association with cosmetic practices and health risk assessment. J. Occup. Med. Toxicol.

[19] Quach T., Nguyen K.D., Doan-Billings P.A., Okahara L., Fan C., Reynolds P. (2008). A preliminary survey of Vietnamese nail salon workers in Alameda County, California. J. Community Health.

[20] Roelofs C., Azaroff L.S., Holcroft C., Nguyen H., Doan T. (2008). Results from a community-based occupational health survey of Vietnamese-American nail salon workers. J. Immigr. Minor. Health.

[21] Reutman S.R., Rohs A.M., Clark J.C., Johnson B.C., Sammons D.L., Toennis C.A., Robertson S.A., MacKenzie B.A., Lockey J.E. (2009). A pilot respiratory health assessment of nail technicians: Symptoms, lung function, and airway inflammation. Am. J. Ind. Med..

[22] Tran H.M., Bui H.T.M., Thoumsang S., Ngo N.T.B., Nguyen N.P.T., Nguyen H.T.M., Nguyen S.M., Hara K., Wangwongwatana S. (2020). Occupational symptoms due to exposure to volatile organic compounds among female Vietnamese nail salon workers in Danang city. J. Occup. Health.

[23] Ma G.X., Wei Z., Husni R., Do P., Zhou K., Rhee J., Tan Y., Navder K., Yeh M.-C. (2019). Characterizing Occupational Health Risks and Chemical Exposures Among Asian Nail Salon Workers on the East Coast of the United States. J. Community Health.

[24] Dang J.V., Rosemberg M.S., Le A.B. (2021). Perceived work exposures and expressed intervention needs among Michigan nail salon workers. Int. Arch. Occup. Environ. Health.

[25] Tran H.M., Bui H.T.M., Thoumsang S., Wangwongwatana S., Nguyen H.P.A., Phung D. (2022). Health risk assessment of volatile organic compounds exposure among nail salon workers in Vietnam. Hum. Ecol. Risk Assess. Int. J..

[26] IARC (2018). Benzene.

[27] U.S. EPA (2003). IRIS Chemical Assessment Summary: Benzene.

[28] IARC (2006). Formaldehyde, 2-Butoxyethanol and 1-Tert-Butoxypropan-2-ol.

[29] U.S. EPA (2024). IRIS Toxicological Review of Formaldehyde (Inhalation).

[30] Keretetse G., Brouwer D., Nelson G. (2022). Evaluating awareness of health risks and self-reported symptoms among nail technicians in Johannesburg, South Africa—A pilot study. Occup. Health S. Afr..

[31] Keretetse G., Nelson G., Brouwer D. (2023). Exposure of formal and informal nail technicians to organic solvents found in nail products. Front. Public Health.

[32] Masekameni M.D., Moolla R., Gulumian M., Brouwer D. (2018). Risk Assessment of Benzene, Toluene, Ethyl Benzene, and Xylene Concentrations from the Combustion of Coal in a Controlled Laboratory Environment. Int. J. Environ. Res. Public Health.

[33] Hoseini L.K., Yengejeh R.J., Rouzbehani M.M., Sabzalipour S. (2022). Health risk assessment of volatile organic compounds (VOCs) in a refinery in the southwest of Iran using SQRA method. Front. Public Health.

[34] Dehghani F., Omidi F., Fallahzadeh R.A., Pourhassan B. (2021). Health risk assessment of occupational exposure to heavy metals in a steel casting unit of a steelmaking plant using Monte-Carlo simulation technique. Toxicol. Ind. Health.

[35] Mo Z., Lu S., Shao M. (2021). Volatile organic compound (VOC) emissions and health risk assessment in paint and coatings industry in the Yangtze River Delta, China. Environ. Pollut..

[36] Cheng C.A., Ching T.C., Tsai S.W., Chuang K.J., Chuang H.C., Chang T.Y. (2022). Exposure and health risk assessment of indoor volatile organic compounds in a medical university. Environ. Res..

[37] Natarajan S., Mukhopadhyay K., Thangaswamy D., Natarajan A., Chakraborty D. (2022). Characterisation of Indoor Volatile Organic Compounds and Its Association with Respiratory Symptoms Among Children Living in Solid Fuel Using Households in Tamil Nadu, India. MAPAN.

[38] Mahdavi V., Omar S.S., Zeinali T., Sadighara P., Fakhri Y. (2023). Carcinogenic and non-carcinogenic risk assessment induced by pesticide residues in fresh pistachio in Iran based on Monte Carlo simulation. Environ. Sci. Pollut. Res. Int..

[39] U.S. EPA (2024). Exposure Assessment Tools by Routes—Inhalation. https://www.epa.gov/expobox/exposure-assessment-tools-routes-inhalation.

[40] U.S. EPA IRIS Assessments. List A to Z. https://iris.epa.gov/AtoZ/?list_type=alpha.

[41] U.S. EPA (2011). Exposure Factors Handbook.

[42] Statistics South Africa (2011). Mid-year Population Estimates, Statistical Release P0302. https://www.statssa.gov.za/publications/P0302/P03022021.pdf.

[43] Burleigh-Flayer H., Garman R., Neptun D., Bevan C., Gardiner T., Kapp R., Tyler T., Wright G. (1997). Isopropanol vapor inhalation oncogenicity study in Fischer 344 rats and CD-1 mice. Fundam. Appl. Toxicol..

[44] Lavoue J. Expostat—Statistical Tools for the Interpretation of Industrial Hygiene Data. https://expostats.ca/site/en/tools.html.

[45] Lavoue J. NDExpo—Treatment of Non-detects in Industrial Hygiene Samples. https://expostats.ca/site/en/othertools.html.

[46] JASP. https://jasp-stats.org/.

[47] ACGIH (2021). 2021 TLVs and BEIs: Based on the Documentation of the Threshold Limit Values for Chemical Substances and Physical Agents & Biological Exposure Indices.

[48] Department of Employment and Labour South Africa (2021). Regulations for Hazardous Chemical Agents, South Africa, 2021. Government Gazette No. 44348. https://www.labour.gov.za/DocumentCenter/Publications/Occupational%20Health%20and%20Safety/Regulations%20for%20Hazardous%20Chemical%20Agents%202021.pdf.

[49] U.S. EPA (2003). IRIS Chemical Assessment Summary: Acetone.

[50] Preller L., Burstyn I., DE Pater N., Kromhout H. (2004). Characteristics of peaks of inhalation exposure to organic solvents. Ann. Occup. Hyg..

[51] Virji M.A., Liang X., Su F.-C., LeBouf R.F., Stefaniak A.B., Stanton M.L., Henneberger P.K., Houseman E.A. (2019). Peaks, Means, and Determinants of Real-Time TVOC Exposures Associated with Cleaning and Disinfecting Tasks in Healthcare Settings. Ann. Work. Expo. Health.

[52] Seo J.Y., Han I., Au E., Li A., Tomas C., Chao Y.Y. (2024). Evaluating Occupational Workforce and Practices in New York Metropolitan Nail Salons. New Solut..

[53] Lamb J., Hesse S., Miller B.G., MacCalman L., Schroeder K., Cherrie J., van Tongeren M. (2015). Evaluation of the Tier 1 Exposure Assessment Models Used Under REACH (Eteam) Project.

