# Supplementary material for: Health Risk Assessment of Nail Technicians in the Formal and Informal Sectors of Johannesburg, South Africa
Source: Int J Environ Res Public Health. 2025 Feb 24;22(3):330. doi: 10.3390/ijerph22030330 (PMC11942596; doi:10.3390/ijerph22030330)
Supplement: Supplementary file 1 [file ijerph-22-00330-s001.zip › ijerph-3405324-supplementary.pdf]

# Supplementary Material

**Table S1.** Questionnaire.

**Title: Occupational exposure to chemicals, and health outcomes, among nail technicians in Johannesburg, South Africa.**

| **Demographic information** | | | | | | | |
|---|---|---|---|---|---|---|---|
| **1. Subject number/code:** | | | | | | | |
| **2. Date:** | | | | | | | |
| **3. Age:** | | | | | | | |
| **4. Sex:** | | **Male** | | **Female** | | | |
| **5. What is your current occupation?** *(Please tick the appropriate box)* | | | | | | | |
| **Nail technicians** | | **Beauty therapist** | | **Hairdresser** | | **Other: (Specify)** ………………………… | |
| **6. Do you have other sources of income other than your current job?** *(Please tick the appropriate box)* | | | | | | **Yes** | **No** |
| | | | | | | | |
| **If so, what are they?** | | | | | | | |
| **7. How long have you been working in this current job?** | | | | | | | |
| **8. What was your previous job?** | | | | | | | |
| **9. How many clients do you see per day in your current job?** | | | | | | | |
| **10. How many hours per day do you work?** *(Please tick the appropriate box)* | | | | | | | |
| 6 hours/day | | | | 12 hours/day | | | |
| 8 hours/day | | | | Other | | | |
| **11. How many days per week do you work?** *(Please tick the appropriate box)* | | | | | | | |
| 5 days/week | | | | | | | |
| 6 days/week | | | | | | | |
| 7 days/week | | | | | | | |
| Other | | | | | | | |

| **Work practices and perception of health risks** | | | | | |
|---|---|---|---|---|---|
| **12. What type of nail applications do you perform and how often?** *(Please tick the appropriate box)* | **Type of Nail application performed** | **Every day** | **Once a week** | **Once a month** | **Once every 3 months** |
| Buff and paint | | | | | |

| | | | | | |
|---|---|---|---|---|---|
| UV gel/gelish | | | | | |
| Acrylic method | | | | | |
| All of the above | | | | | |
| **13. What type of nail products do you use and how often?** *(Please tick the appropriate box)* | **Type of Nail Product used** | **Every day** | **Once a week** | **Once a month** | **Once every 3 months** |
| Nail polish | | | | | |
| Nail polish remover | | | | | |
| Liquid and powder acrylic | | | | | |
| UV gel | | | | | |
| Primer | | | | | |
| Nail tips glue | | | | | |
| Artificial nail tips remover | | | | | |
| All of the above | | | | | |

| | | |
|---|---|---|
| **14. Have you ever read the label of these nail products?** *(Please tick the appropriate box)* | **Yes** | **No** |
| | | |
| **15. Do you know the names of the chemicals contained in nail products that you use?** *(Please tick the appropriate box)* | **Yes** | **No** |
| Acetone | | |
| Ethanol | | |
| Ethyl acetate | | |
| Ethyl methacrylate | | |
| Methyl methacrylate | | |
| n-Butylacetate | | |
| Toluene | | |
| Xylene | | |
| Dibutyl phthalate | | |
| Formaldehyde | | |
| Isopropyl acetate | | |

| | | |
|---|---|---|
| Titanium dioxide | | |

| **16. How harmful do you think these nail products are?** *(Please tick the appropriate box)* | **Not at all** | **Slightly** | **Moderately** | **Extremely** |
|---|---|---|---|---|
| Nail polish | | | | |
| Nail polish remover | | | | |
| Liquid and powder acrylic | | | | |
| UV gel | | | | |
| Primer | | | | |
| Nail tips glue | | | | |
| Artificial nail tips remover | | | | |

| **17. Have you received any training on the effects of these nail products and chemicals?** *(Please tick the appropriate box)* | **Yes** | **No** |
|---|---|---|
| | | |
| **If yes then how long was the training?** | | |

| **18. Do you take any steps to protect yourself from the effects of these chemicals?** *(Please tick the appropriate box)* | **Yes** | **No** |
|---|---|---|
| | | |

| **19. If yes, how do you protect yourself?** *(Please tick as many as applicable)* | |
|---|---|
| Extraction ventilation (ceiling extraction fan, table extraction fan, etc.) | |
| Dilution ventilation (opening doors) | |
| Use gloves | |
| Use mask | |
| Use goggles | |
| Avoid skin contact | |
| Put lids on containers | |
| Keep lids on containers | |
| Decant products into smaller pots | |
| Good hygiene | |
| Use product as instructed | |

| **20. If not, is there a reason why you don't?**<br>***(Please tick as many as applicable)*** | | |
|---|---|---|
| Not needed | | |
| Products are safe | | |
| Product considered low risk | | |
| No fumes with this product | | |
| Has never caused harmed before | | |
| Mask gets in the way | | |
| Mask uncomfortable/off-putting | | |
| Difficulty using gloves | | |
| Nothing to protect ourselves with | | |
| No reason | | |
| **21. Have you experienced any ill-health symptoms since working as a nail technician?**<br>***(Please tick the appropriate box)*** | **Yes** | **No** |
| | | |

| **22. If yes, which ill-health symptoms have you suffered from?**<br>***(Please tick as many as applicable)*** | | |
|---|---|---|
| *Symptom 1* | Headaches | |
| *Symptom 2* | Light-headedness | |
| *Symptom 3* | Difficulty breathing | |
| *Symptom 4* | Chest tightness | |
| *Symptom 5* | Regular cough | |
| *Symptom 6* | Wheezing | |
| *Symptom 7* | Nasal congestion | |
| *Symptom 8* | Doctor diagnosed asthma | |
| *Symptom 9* | Throat irritation | |
| *Symptom 10* | Nose irritation | |
| *Symptom 11* | Eye-irritation | |
| *Symptom 12* | Skin problems (dryness, scaling, rash, redness) | |
| *Symptom 13* | Eczema | |

| | | |
|---|---|---|
| *Symptom 14* | Neck pain | |
| *Symptom 15* | Shoulder pain | |
| *Symptom 16* | Wrist and hand pain | |
| *Symptom 17* | Upper back pain | |
| *Symptom 18* | Lower back pain | |
| *Symptom 19* | Leg or feet pain | |

**23. Have you suffered from any of the following ill-health symptoms in the last 4 weeks?**
***(Please tick the appropriate box)***

| ***Symptom*** | **Yes** | **No** | ***Symptom*** | **Yes** | **No** |
|---|---|---|---|---|---|
| *Symptom 1* | | | *Symptom 11* | | |
| *Symptom 2* | | | *Symptom 12* | | |
| *Symptom 3* | | | *Symptom 13* | | |
| *Symptom 4* | | | *Symptom 14* | | |
| *Symptom 5* | | | *Symptom 15* | | |
| *Symptom 6* | | | *Symptom 16* | | |
| *Symptom 7* | | | *Symptom 17* | | |
| *Symptom 8* | | | *Symptom 18* | | |
| *Symptom 9* | | | *Symptom 19* | | |
| *Symptom 10* | | | | | |

**24. Do any of these symptoms get better when you are away from work for more than one day?**
***(Please tick the appropriate box)***

| ***Symptom*** | **Yes** | **No** | ***Symptom*** | **Yes** | **No** |
|---|---|---|---|---|---|
| *Symptom 1* | | | *Symptom 11* | | |
| *Symptom 2* | | | *Symptom 12* | | |
| *Symptom 3* | | | *Symptom 13* | | |
| *Symptom 4* | | | *Symptom 14* | | |
| *Symptom 5* | | | *Symptom 15* | | |
| *Symptom 6* | | | *Symptom 16* | | |
| *Symptom 7* | | | *Symptom 17* | | |

| | | | | | |
|---|---|---|---|---|---|
| *Symptom 8* | | | *Symptom 18* | | |
| *Symptom 9* | | | *Symptom 19* | | |
| *Symptom 10* | | | | | |

**Table S2.** Categories of self-reported symptoms.

| | **Category Name** | **Self-reported symptoms** |
|---|---|---|
| Category A | Neurological effects | Headache |
| | | Light-headedness |
| Category B | Respiratory effects | Difficulty breathing |
| | | Chest tightness |
| | | Regular cough |
| | | Wheezing |
| | | Nasal congestion |
| | | Doctor diagnosed asthma |
| | | Throat irritation |
| | | Nose irritation |
| Category C | Eye irritation | Eye irritation |
| Category D | Skin irritation | Skin problems |
| | | Eczema |

**Table S3.** Volatile Organic Compounds categories according to the similarity of health effects.

| **Category A Neurologic effects** | **Category B Respiratory effects** | **Category C Eye irritation** |
|---|---|---|
| Ethanol | Ethanol | Ethanol |
| Acetone | Acetone | Acetone |
| Benzene | Ethyl Acetate | Ethyl Acetate |
| Propyl Acetate | Benzene | Benzene |
| Toluene | MMA | MMA |
| n-Butyl Acetate | EMA | EMA |
| Xylene | Propyl Acetate | Propyl Acetate |
| 2-Propanol | Toluene | Toluene |
| White spirit | n-Butyl Acetate | n-Butyl Acetate |
| | Xylene | Xylene |
| | 2-Propanol | 2-Propanol |
| | White spirit | White spirit |
| | Formaldehyde | Formaldehyde |

**Table S4.** Correlation coefficient and p-values between self-reported symptoms and VOC concentrations among formal and informal nail technicians.

| | Formal nail technicians | | | | | | | | Informal nail technicians | | | | | | | |
|---|---|---|---|---|---|---|---|---|---|---|---|---|---|---|---|---|
| | Neurological Effects | | Respiratory Effects | | Eye irritation | | Symptom score | | Neurological Effects | | Respiratory Effects | | Eye irritation | | Symptom score | |
| | *rho* | *p-value* | *rho* | *p-value* | *rho* | *p-value* | *rho* | *p-value* | *rho* | *p-value* | *rho* | *p-value* | *r* | *p-value* | *r* | *p-value* |
| Age | 0.291 | 0.414 | 0.491 | 0.150 | 0.608 | 0.062 | 0.491 | 0.150 | -0.216 | 0.550 | **-0.814**[a] | 0.004[b] | -0.114 | 0.753 | -0.489 | 0.151 |
| Sex | - | - | - | - | - | - | - | - | **-0.797**[a] | 0.006[b] | -0.080 | 0.827 | -0.218 | 0.545 | -0.508 | 0.134 |
| | *r* | *p-value* | *r* | *p-value* | *r* | *p-value* | *r* | *p-value* | *r* | *p-value* | *r* | *p-value* | *r* | *p-value* | *r* | *p-value* |
| Work experience | 0.118 | 0.745 | 0.127 | 0.726 | 0.377 | 0.283 | 0.259 | 0.471 | -0.388 | 0.268 | -0.359 | 0.308 | -0.393 | 0.261 | -0.477 | 0.163 |
| Clients serviced | -0.072 | 0.843 | 0.342 | 0.333 | 0.502 | 0.139 | 0.296 | 0.407 | -0.328 | 0.355 | -0.012 | 0.974 | -0.135 | 0.711 | -0.221 | 0.540 |
| Working hours/day | -0.351 | 0.321 | -0.435 | 0.208 | -0.373 | 0.289 | -0.418 | 0.229 | 0.304 | 0.393 | **0.931**[b] | <0.001 | 0.535 | 0.111 | **0.657**[b] | 0.039 |
| Cat A VOC | -0.349 | 0.322 | - | - | - | - | - | - | -0.437 | 0.207 | - | - | - | - | - | - |
| Cat B VOC | - | - | -0.067 | 0.853 | - | - | - | - | - | - | 0.131 | 0.718 | - | - | - | - |
| Cat C VOC | - | - | - | - | -0.162 | 0.655 | - | - | - | - | - | - | -0.343 | 0.332 | - | - |
| $ACGIH_{index}$ | -0.356 | 0.312 | -0.137 | 0.706 | -0.261 | 0.466 | -0.297 | 0.405 | -0.032 | 0.931 | -0.033 | 0.928 | -0.218 | 0.545 | -0.148 | 0.684 |

Cat A VOC, VOCs with neurological effects; Cat B VOC, VOCs with respiratory effects; Cat C VOC, VOCs with eye irritation effects; $ACGIH_{index}$, additive respiratory effects. [a]represents the point-biserial correlation $r_{pb}$. [b] Statistically significant ($p < 0.05$).
